# Chemerin Added to Endothelin-1 Promotes Rat Pulmonary Artery Smooth Muscle Cell Proliferation and Migration

**DOI:** 10.3389/fphys.2020.00926

**Published:** 2020-07-30

**Authors:** Aliénor Hanthazi, Pascale Jespers, Grégory Vegh, Christine Dubois, Géraldine Hubesch, Jean-Yves Springael, Laurence Dewachter, Kathleen Mc Entee

**Affiliations:** ^1^Laboratory of Physiology and Pharmacology, Faculty of Medicine, Université libre de Bruxelles, Brussels, Belgium; ^2^Laboratory of Stem Cells and Cancer, Faculty of Medicine, Université libre de Bruxelles, Brussels, Belgium; ^3^Institute of Interdisciplinary Research (IRIBHM), Faculty of Medicine, Université libre de Bruxelles, Brussels, Belgium

**Keywords:** CMKRL1, chemerin, endothelin-1, pulmonary artery, smooth muscle cells, proliferation, migration, apoptosis

## Abstract

**Background:**

While chemerin has been shown to increase proliferation and migration of systemic vascular smooth muscle cells (SMCs) contributing therefore to the development of hypertension, this remains to be clarified for the pulmonary circulation.

**Methods:**

Expression of chemerin and its three receptors (CMKRL1, CCRL2, GPR1) was examined by immunohistochemistry and RTq-PCR in lungs, pulmonary artery, and thoracic aorta from Wistar rats. Primary cultured rat pulmonary artery and thoracic aorta SMCs treated with recombinant chemerin (tested from 5.10^–9^ to 10^–7^ mol/L) were assessed for proliferation and migration (both with 10^–7^ mol/L endothelin-1), as well as for staurosporine-induced apoptosis.

**Results:**

In pulmonary artery and thoracic aorta, CMKLR1 expression was detected in both endothelial cells and SMCs. In primary cultured pulmonary artery SMCs, chemerin and its three receptors were expressed, and CMKLR1 expression was higher than those of CCRL2 and GPR1. Chemerin added to endothelin-1 increased pulmonary artery SMC proliferation, while chemerin or endothelin-1 alone did not. This effect was less pronounced in thoracic aorta SMCs. Chemerin induced pulmonary artery and thoracic aorta SMC migration, which was exacerbated by endothelin-1 and more pronounced in thoracic aorta SMCs. Chemerin concentration-dependently reduced staurosporine-induced apoptosis in both pulmonary artery and thoracic aorta SMCs. In pulmonary artery SMCs, endothelin-1 treatment increased the expression of CMKLR1, CCRL2, and GPR1, while these expressions were not altered in thoracic aorta SMCs.

**Conclusion:**

Chemerin/CMKRL1 signaling, in conjunction with a key mediator in the pathogenesis of pulmonary hypertensive diseases, endothelin-1, stimulated proliferation and migration, and increased resistance to apoptosis in rat primary cultured pulmonary artery SMCs. Our results suggest that this signaling could play a role in pulmonary artery remodeling observed in pulmonary hypertension.

## Introduction

Pulmonary hypertension due to left heart disease (or group 2 pulmonary hypertension), which is often associated with metabolic syndrome, is the most prevalent form of pulmonary hypertension worldwide (Friedman and Andrus, 2012; Vachiery et al., 2019). Inflammation associated with metabolic syndrome, notably elevated pro-inflammatory cytokines, is known to contribute to adverse pulmonary vascular remodeling observed in group 2 pulmonary hypertension (Weiss et al., 2013; Ranchoux et al., 2019). Adipokines and cytokines have also been incriminated in the pathogenesis of pulmonary artery remodeling in pulmonary arterial hypertension, independently of obesity or metabolic syndrome (Summer et al., 2009; Huertas et al., 2012; Savai et al., 2012).

Initially described as a regulator of energetic metabolism and inflammatory/immune responses (Wittamer et al., 2003; Bozaoglu et al., 2007; Goralski et al., 2007), the adipokine chemerin was identified as a key mediator regulating systemic vascular homeostasis and tone, and contributing therefore to the pathogenesis of hypertension (Watts et al., 2013). Chemerin levels have been recently associated with the development of intimal hyperplasia in aortic tissue, suggesting a role played by chemerin in atherosclerotic plaque formation (Liu et al., 2019). Chemerin and its main receptor, the chemerin chemokine-like receptor 1 (CMKRL1), are both expressed by vascular cells and have been incriminated in cardiovascular pathophysiological processes, such as atherosclerosis (Kostopoulos et al., 2014).

Chemerin, which mainly acts through the activation of CMKLR1 (Kennedy et al., 2016), was identified in the *tunica intima* and the *tunica media* of systemic (Watts et al., 2013) and pulmonary arteries (Hanthazi et al., 2019). Chemerin has been shown to promote proliferation and migration of myocytes (Yang et al., 2012) or vascular smooth muscle cells (SMCs), contributing to carotid intimal hyperplasia (Kunimoto et al., 2015). Chemerin also potently increased production and release of pro-inflammatory cytokines such as tumor necrosis factor (TNF)-α, interleukin (IL)-1, and IL-6 (Kaur et al., 2010) contributing to a sustained inflammatory phenotype. Circulating chemerin levels were also elevated in chronic inflammatory diseases and states, associated or not with obesity (Kukla et al., 2010; Nakajima et al., 2010) and correlated to systemic levels of pro-inflammatory cytokines, such as TNF-α, IL-6, and C-reactive protein (Lehrke et al., 2009; Weigert et al., 2010).

In this context, we hypothesized that chemerin (alone or in association with other factors already known to be implicated in the pathogenesis of pulmonary hypertension) may influence pulmonary artery SMC proliferation, apoptosis, and migration, which are crucial to the pathophysiological development of pulmonary artery remodeling observed in pulmonary hypertensive diseases.

## Materials and Methods

### Animals

All procedures involving animals were performed in accordance with ethical standards and were approved by the Institutional Animal Care and Use Committee of the Faculty of Medicine at the Université libre de Bruxelles (Brussels, Belgium; protocol acceptation number: 561N). Applicable guidelines were followed in accordance with the “Guide for the Care and Use of Laboratory Animals” published by the US National Institutes of Health (NIH publication no. 85–23, revised 1996).

After a one-week-acclimatization period, male Wistar rats weighting 504 ± 9 g (Janvier, Le Genest-Saint-Isle, France) were euthanized with carbon dioxide. Artery sections were then used for primary culture of vascular cells and lungs, and artery segments were embedded in paraffin, after 24-h fixation in 10%-neutral buffered formalin, for immunofluorescent labeling.

### Primary Culture and Treatment of Rat Pulmonary Artery and Thoracic Aorta SMCs

Pulmonary artery and thoracic aorta SMCs were isolated and cultured using the explant technique, as previously described (Eddahibi et al., 2001). Briefly, after euthanasia of the animals, thoracic aorta and pulmonary artery were excised under sterile conditions and kept in Dulbecco’s Modified Eagle Medium (DMEM; Gibco, Paisley, United Kingdom) at 4°C to strip off their intimal and residual adventitial layers. Dissected media was cut into small pieces, which were transferred into cell-culture plates (Sarstedt, Nümbrecht, Germany). Artery fragments were incubated in DMEM supplemented with 10% fetal calf serum (FCS; Gibco), 25 mmol/L HEPES (Gibco), an anti-mycotic agent (250 μg/mL Fungizone; Gibco) and antibiotics (10000 U/mL and 10000 μg/mL of penicillin/streptomycin; Gibco, New York, United States) to allow vascular SMCs to grow out. When cell cultures reached 90% confluence, SMCs were detached with 0.05% Trypsin-EDTA (Gibco), collected in culture medium and transferred into new cell-culture flasks. Primary cultured pulmonary artery and thoracic aorta SMCs were used between passages 3 and 6. Phenotype of primary cultured rat pulmonary artery and thoracic aorta SMCs was assessed by the expression of muscle-specific contractile and cytoskeletal proteins using immunofluorescence staining (Eddahibi et al., 2001).

Primary cultured rat pulmonary artery and thoracic aorta SMCs were plated in fresh 10% FCS supplemented DMEM, allowed to grow to 80% confluence and harvested for the evaluation of chemerin and receptor expression. For chemerin or endothelin-1 *in vitro* treatment, pulmonary artery and thoracic aorta SMCs were placed in serum-free medium for 24 h and exposed to increasing concentrations of recombinant mouse chemerin (5.10^–9^, 10^–8^, 5.10^–8^, and 10^–7^ mol/L; aa 17–156; R&D Systems, Minneapolis, MN, United States) and/or endothelin-1 (10^–7^ mol/L, Sigma-Aldrich, St Louis, MO, United States) for 5 h. Tested concentrations of chemerin corresponded to previously reported physiological (normotensive normal rats) and pathological (obese and diabetic rats) blood levels of chemerin (Stelmanska et al., 2013; Lin et al., 2019).

### Real-Time Quantitative Polymerase Chain Reaction (RTq-PCR)

Total RNA was extracted from trypsinized SMCs using RNeasy^®^ Mini Kit (Qiagen, Hilden, Germany), according to the manufacturer’s instructions. RNA concentration was determined using spectrophotometry with a Nanodrop^®^ ND-1000 (Isogen Life Science, De Meern, Netherlands), and RNA integrity was assessed by visual inspection of GelRed (Biotium, Hayward, CA, United States)-stained agarose gels. Reverse transcription was performed with SuperScript^TM^ II Reverse Transcriptase (Invitrogen by Thermo Fisher Scientific, Merelbeke, Belgium), according to the manufacturer’s instructions (Hanthazi et al., 2019).

For RTq-PCR, sense and anti-sense primers were designed, using Primer3 program, for *rattus norvegicus* chemerin (also called RARRES2), CMKLR1 (also called ChemR23), CCRL2, GPR1, regulators of apoptosis Bax (pro-apoptotic) and Bcl2 (anti-apoptotic), endothelin receptors type A and B, interleukin (IL)-6 and its receptor IL-6R, IL-1β, tumor necrosis factor (TNF)-α, glyceraldehyde 3-phosphate dehydrogenase (GAPDH) and hypoxanthine phosphoribosyltransferase (HPRT) 1 mRNA sequence (Table 1 and Supplementary Table S1). Intron-spanning primers were selected and a BLAST analysis was run to check if primer pairs were only matching the sequence of interest. For each sample, amplification reaction was performed in triplicate using SYBRGreen PCR Master Mix (Quanta Biosciences, Gaithersburg, MD, United States), specific primers, and diluted template cDNA. Result analysis was performed using an iCycler system (BioRad Laboratories, Nazareth Eke, Belgium). Relative quantification was achieved with Pfaffl method (Pfaffl, 2001) by normalization with the housekeeping genes, GAPDH and HPRT1 (Hanthazi et al., 2019). Results were expressed as relative fold increase over the mean value of relative mRNA expression of chemerin or CMKLR1 in pulmonary artery SMCs arbitrarily fixed to 1.

**TABLE 1:** Primers used for real-time quantitative polymerase chain reaction (RTq-PCR) in *rattus norvegicus* samples.

Genes		Sequences
**Chemerin** (RARRES2)	Sense; Antisense	5′-AAGGACTGGAAAAAGCCAGAG-3′; 5′-TCCGGCCTAGAACTTTACCC-3′
Chemokine-like receptor 1 (**CMKLR1** or ChemR23)	Sense; Antisense	5′-GACCGGATTAGAACCCCAGT-3′; 5′-AAAACCCCAAACCCATTAGC-3′
C-C chemokine receptor-like 2 (**CCRL2**)	Sense; Antisense	5′-ACCACGCTGTTTTAGGCAGT-3′; 5′-TGGGTCTGTCCCACTGTTGTC-3′
G protein-coupled receptor 1 (**GPR1**)	Sense; Antisense	5′-GGAGCTCAGCATTCATCACA-3′; 5′-GACAGGCTCTTGGTTTCAGC-3′
Bcl2 associated X (**Bax**)	Sense; Antisense	5′-CGTGGTTGCCCTCTTCTACT-3′; 5′-TCACGGAGGAAGTCCAGTGT-3′
Bcl2 apoptosis regulator (**Bcl2**)	Sense; Antisense	5′-TTTCTCCTGGCTGTCTCTGAA-3′; 5′-CATATTTGTTTGGGGCAGGT-3′
Endothelin receptor type A (**ETA**)	Sense; Antisense	5′-CTGGTGGCTCTTTGGATTCT-3′; 5′-GCTCCCATTCCTTCTGTTGA-3′
Endothelin receptor type B (**ETB**)	Sense; Antisense	5′-CGATTGTATCATGCCTCGTG-3′; 5′-GGGACCATTTCTCATGCACT-3′
Glyceraldehyde-3-phosphate dehydrogenase (GAPDH)	Sense; Antisense	5′-AAGATGGTGAAGGTCGGTGT-3′; 5′-ATGAAGGGGTCGTTGATGG-3′
Hypoxanthine phosphoribosyltransferase 1 (HPRT1)	Sense; Antisense	5′-ACAGGCCAGACTTTGTTGGA-3′; 5′-TCCACTTTCGCTGATGACAC-3′

### Immunofluorescent Staining

Three-micrometer pulmonary tissue, pulmonary artery, and thoracic aorta sections were dewaxed and progressively rehydrated in alcoholic baths. Nonspecific binding sites were blocked with normal horse serum [1:20 diluted in phosphate buffered saline (PBS); Vector Laboratories, Burlingame, CA, United States]. After overnight incubation with a polyclonal rabbit anti-rat CMKLR1 (1:100 diluted in PBS; Bioss Antibodies, Aachen, Germany) and a polyclonal goat anti-rat CD31 (1:20 diluted in PBS; R&D Systems, Minneapolis, MN, United States) or a polyclonal goat anti-rat α-smooth muscle actin (1:100 diluted in PBS; Abcam, Cambridge, United Kingdom) primary antibodies and rabbit IgG (1:10000 diluted in PBS; Vector Laboratories; used as negative control), sections were incubated for 1 h with corresponding secondary fluorescent-labeled antibodies [Alexa Fluor^TM^ 488-conjugated donkey anti-rabbit IgG (1:200 diluted in PBS; Thermo Fisher Scientific, Rockford, IL, United States) and Alexa Fluor^TM^ 594-conjugated donkey anti-goat IgG (1:200 diluted in PBS; Invitrogen by Thermo Fisher Scientific, Eugene, OR, United States)]. Nuclei were counterstained with 4’,6-diamidino-2-phenylindole (DAPI) and mounting was performed using ProLong Gold antifade reagent with DAPI (Invitrogen by Thermo Fisher Scientific). Rat spleen sections were used as positive controls for CMKLR1 immunofluorescent labeling. Images were taken using a fluorescence microscope (Leica DM2000, Leica Microsystems, Wetzlar, Germany).

### Cell Proliferation Assay

Proliferation of primary cultured pulmonary artery and thoracic aorta SMCs was assessed using a colorimetric Cell Proliferation ELISA (Roche, Mannheim, Germany), evaluating 5-bromo-2’-deoxyuridine (BrdU) incorporation during DNA synthesis in replicating cells. Briefly, pulmonary artery and thoracic aorta SMCs in DMEM supplemented with 10% FCS were seeded in 96-well plates at a density of 104 cells/well and allowed to adhere for 24 h. Cells were subjected to 24 h of growth arrest in FCS-free DMEM and then treated with increasing concentrations of recombinant mouse chemerin (5.10^–9^, 10^–8^, 5.10^–8^, and 10^–7^ mol/L) with or without endothelin-1 (10^–7^ mol/L) in FCS-free DMEM and diluted BrdU labeling reagent (1:100). After a 24-h incubation period, BrdU incorporation was revealed using an anti-BrdU-peroxidase antibody, according to manufacturer’s recommendations (Cell Proliferation ELISA, Roche) and absorbance was measured using a microplate reader (Packard, Canberra, Australia) at 370 nm. Proliferation levels were expressed as relative fold increase over the mean value of the proliferative rate of the basal condition (corresponding to FCS-free DMEM) fixed to 0.

### Cell Migration Assay

Pulmonary artery and thoracic SMC migration was assessed with FluoroBlok^TM^ colored PET membrane inserts for 24-well plates with 8.0 μm pores (Corning, Durham, NC, United States), according to the manufacturer’s instructions. Briefly, SMCs in FCS-free DMEM were loaded, in the upper insert compartment of 24-well plates, at a density of 25.10^4^ cells/well. In the lower insert compartment, wells were filled with FCS-free DMEM containing increasing concentrations of recombinant mouse chemerin (5.10^–9^, 10^–8^, 5.10^–8^, and 10^–7^ mol/L) in the presence or absence of endothelin-1 (10^–7^ mol/L). After a 24-h incubation period, SMCs were fixed with methanol (Merck, Darmstadt, Germany) for 15 min, and nuclei were stained with DAPI (1:10000 diluted in PBS) for 5 min. Between each step, cells were washed carefully with PBS. Fluorescently labeled cells having migrated through the membrane were detected by fluorescence microscopy (Leica DM2000, Leica Microsystems, Wetzlar, Germany). For each experimental condition, three inserts were seeded and five randomly chosen fields were evaluated at a 10× magnitude after acquisition of contrasted fluorescence microscopy images. Mean cell number for each condition was obtained by calculation of a mean cell number per five fields evaluated by two independent operators in a blinded manner.

### Cell Apoptosis Assay

In pulmonary artery and thoracic aorta SMCs, apoptosis was evaluated by FACS analysis using Bio Legend’s FITC Annexin V Apoptosis Detection Kit with propidium iodide (Bio Legends, San Diego, CA, United States). Briefly, cultured SMCs were seeded in 10% FCS-supplemented DMEM and allowed to grow to 80% confluence. Medium was then removed, and cells were subjected to growth arrest in presence of FCS-free DMEM for 24 h. After, apoptosis was induced by staurosporine (10^–6^ mol/L; Sigma-Aldrich) in the presence of increasing concentrations of recombinant mouse chemerin (5.10^–9^, 10^–8^, 5.10^–8^, and 10^–7^ mol/L) for 24 h. Floating cells were collected and combined with adherent cells harvested by trypsin/EDTA treatment. After centrifugation, SMCs were double-stained with annexin V-FITC and propidium iodide according to the manufacturer’s instructions. In each sample, at least 100 000 cells were counted by FACS analysis (Becton, Dickinson and Company, BD Biosciences, San Jose, CA, United States). The percentage of early (annexin V-positive and propidium iodide-negative cells) and late (annexin V-positive and propidium iodide-positive cells) apoptotic cells was quantified by flow cytometry and considered as the percentage of apoptotic cells.

### Statistical Analysis

Results are presented as mean ± standard error of the mean (SEM). Data were analyzed with a two-factor (groups, doses) analysis of variance (ANOVA) for missing data with repeated measurements on concentrations and interaction, using the algorithm described by Winer et al. (1991).

Inter-group differences of RTq-PCR results were tested with one-way ANOVA. When the *F* ratio of this analysis reached a critical *p*-value < 0.05, comparisons were made with a parametric Student’s *t*-test. A *p*-value < 0.05 was considered as statistically significant; *n* represents the number of rats included.

## Results

### Expression of Chemerin and Its Receptors in Rat Pulmonary Artery and Thoracic Aorta SMCs

**FIGURE 1:**
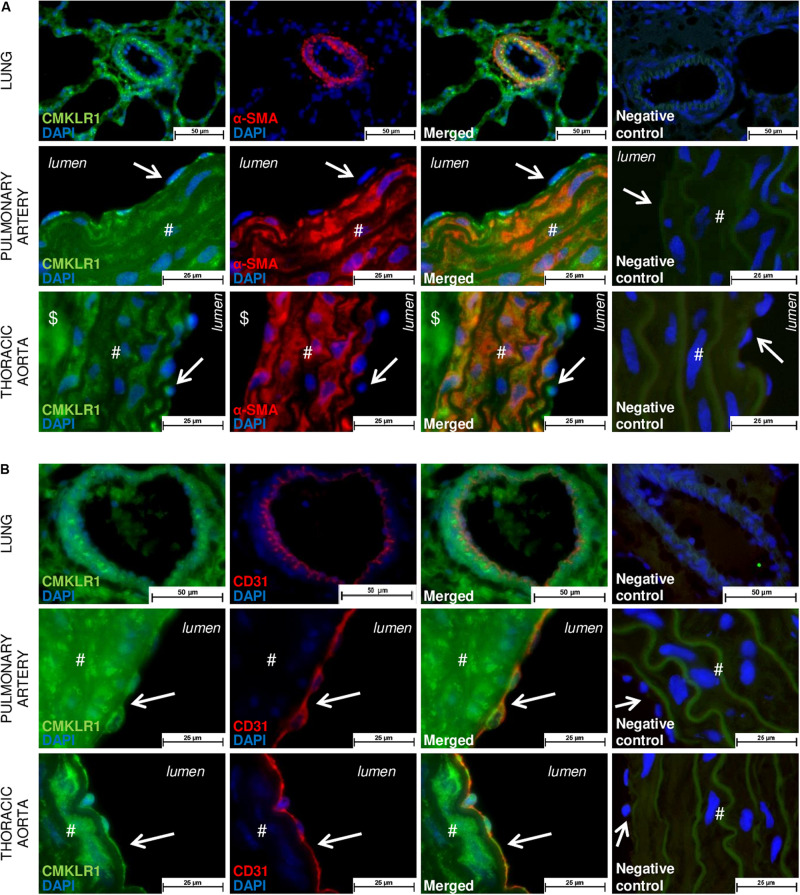
Localization of CMKLR1 in rat pulmonary artery and thoracic aorta. Representative images of double immunofluorescent labeling of CMKLR1 (green) with the smooth muscle-specific marker [red, **(A)**] and with the endothelial-specific marker CD31 [red, **(B)**] in lungs, as well as in pulmonary artery and thoracic aorta for a total of six fields randomly chosen along each type of tissue from Wistar rats (*n* = 4) (both positive for elastic fibers observed in green). Negative control images resulted from the overlay of red, green, and blue (DAPI) channels. Labeling in the vascular intimal, medial, and adventitial layers was, respectively, indicated by arrows, #, and $ in both types of vessels. Scale bars = 50 μm for the lung and 25 μm for the pulmonary artery and thoracic aorta sections.

To localize and identify vascular cells expressing the main activity-linked chemerin receptor, we co-immunostained lung tissues, as well as isolated proximal pulmonary artery and thoracic aorta from Wistar rats, with either antibodies against CMKLR1 together with an endothelial-specific marker CD31 or a smooth muscle-specific marker, the α-smooth muscle actin. In the lungs, microscopic analysis revealed a strong staining of CMKLR1 within the media, more precisely in SMCs of distal pulmonary arteries (Figure 1A). As illustrated in Figures 1A,B, staining of CMKLR1 was weaker in endothelial compared to smooth muscle layer. In proximal pulmonary artery and thoracic aorta, CMKLR1 staining was observed in both medial (Figure 1A) and intimal (Figure 1B) layers, more precisely in pulmonary artery and thoracic aorta smooth muscle (Figure 1A) and endothelial cells (Figure 1B).

**FIGURE 2:**
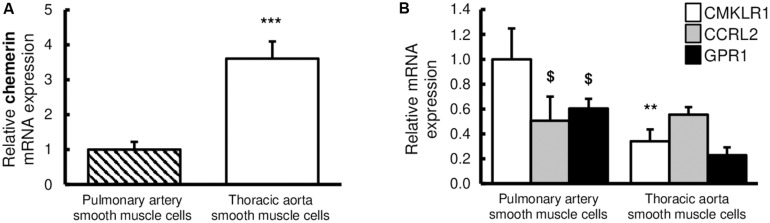
Expressions of chemerin and its receptors in rat pulmonary artery and thoracic aorta. Relative gene expression of chemerin **(A)** and its three receptors **(B)**, the chemokine-like receptor 1 (CMKLR1 also called ChemR23; white bars), the C–C chemokine-like receptor 2 (CCRL2; gray bars), and the G protein-coupled receptor 1 (GPR1; black bars) in primary cultured pulmonary artery (seven experiments in total) and thoracic aorta (eight experiments in total) smooth muscle cells isolated from Wistar rats (*n* = 5). Quantification of relative gene expression was achieved by real-time quantitative polymerase chain reaction (RTq-PCR) using the Pfaffl method with normalization with the housekeeping genes, GAPDH and HPRT1. Results were expressed as relative fold increase over the mean value of relative mRNA expression of chemerin **(A)** and CMKLR1 **(B)** in pulmonary artery smooth muscle cells arbitrarily fixed to 1 and presented as mean ± SEM. **0.001 < *p* < 0.01, ***p < 0.001 compared to the expression of the same gene in pulmonary artery; ^$^0.01 < *p* < 0.05 compared to the gene expression of CMKLR1 in the same type of vessel.

These *in situ* observations were confirmed *in vitro*, by the evaluation of relative gene expressions of chemerin and its receptors, in primary cultured rat pulmonary artery and thoracic aorta SMCs. Indeed, CMKLR1, chemerin, and its two other receptors CCRL2 and GPR1 were all detected in primary cultured rat pulmonary artery and thoracic aorta SMCs (Figures 2A,B). Relative gene expression of chemerin was higher in thoracic aorta compared to pulmonary artery SMCs (Figure 2A). In thoracic aorta SMCs, gene expression levels of CMKLR1, CCRL2, and GPR1 were similar (Figure 2B), while the expression of the main chemerin receptor CMKLR1 was higher than CCRL2 and GPR1 in pulmonary artery SMCs (Figure 2B). Gene expression of CMKRL1 was higher in pulmonary artery than in thoracic aorta SMCs (Figure 2B).

### Chemerin Added to Endothelin-1 Increases SMC Proliferation

**FIGURE 3:**
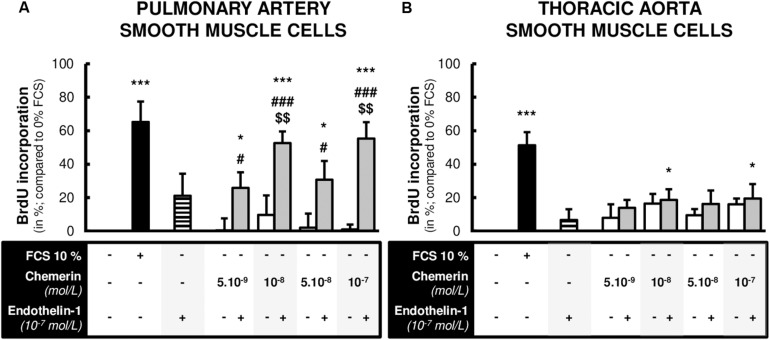
Combined chemerin and endothelin-1 treatment increased the proliferation of pulmonary artery smooth muscle cells. Quantification of 5-bromo-2’-deoxyuridine (BrdU) incorporation in primary cultured pulmonary artery [**(A)**, six experiments in total] and thoracic aorta [**(B)**, seven experiments in total] smooth muscle cells isolated from Wistar rats (*n* = 4), treated or not with endothelin-1 (10^–7^ mol/L) with increasing concentrations of recombinant mouse chemerin (tested from 5.10^–9^ to 10^–7^ mol/L) for 24 h. The proliferative responses were expressed as relative fold increase over the mean value of the proliferative rate of basal condition [corresponding to 0% fetal calf serum (FCS)-treated] in the same type of smooth muscle cells. Results were presented as mean ± SEM. *0.01 < *p* < 0.05 and ***p < 0.001 compared to corresponding 0% FCS-treated cells; ^#^0.01 < *p* < 0.05 and ^###^*p* < 0.001 compared to corresponding chemerin-treated cells; ^$$^0.001 < *p* < 0.01 compared to corresponding endothelin-1-treated cells.

To determine whether chemerin influenced SMC proliferation, primary cultured pulmonary artery and thoracic aorta SMCs were subjected to chemerin alone or in the presence of a well-known factor implicated in the pathogenesis of pulmonary hypertension, the endothelin-1. In pulmonary artery and thoracic aorta SMCs, 24-h treatment with increasing concentrations of chemerin (5.10^–9^, 10^–8^, 5.10^–8^, and 10^–7^ mol/L) or with endothelin-1 (10^–7^ mol/L) alone did not induce any proliferation compared to FCS-free medium (Figure 3). In pulmonary artery SMCs, combined treatment of chemerin and endothelin-1 increased proliferation rates compared to those observed with chemerin alone. This was observed for all tested concentrations of chemerin (Figure 3A). Compared to endothelin-1 alone, combined chemerin and endothelin-1 treatment increased proliferation rate from 10^–8^ mol/L (Figure 3A). In contrast, combined treatment of chemerin and endothelin-1 increased thoracic aorta SMC proliferation rates compared to FCS-free medium but not compared to chemerin or endothelin-1 alone (Figure 3B).

### Chemerin Prevented Staurosporine-Induced Apoptosis in SMCs

**FIGURE 4:**
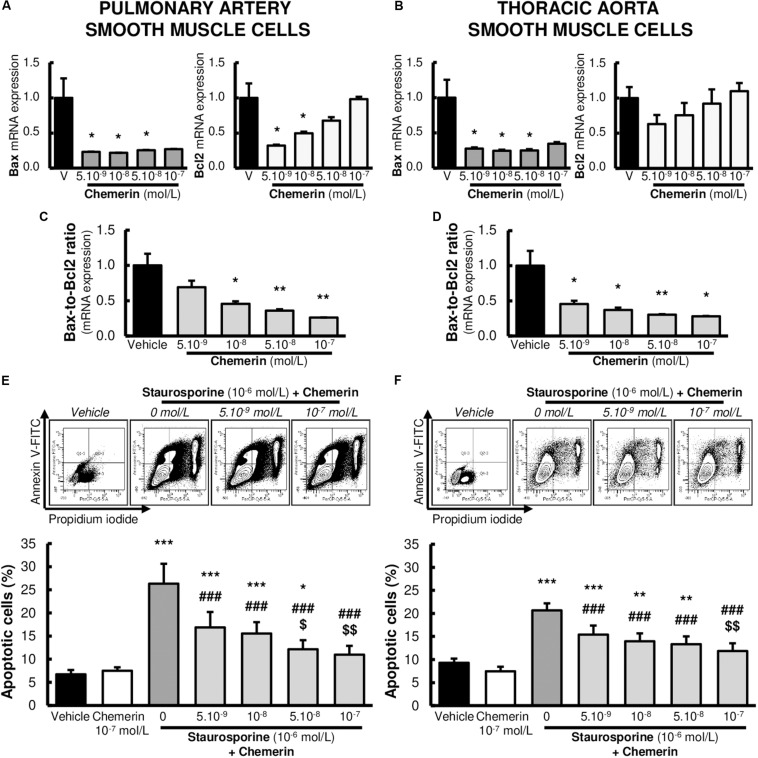
Chemerin decreased the activation of apoptosis in pulmonary artery and thoracic aorta smooth muscle cells. Relative gene expressions of Bax, Bcl2, and pro-apoptotic Bax-to-Bcl2 ratio in primary cultured pulmonary artery [**(A,C)**. six experiments in total] and thoracic aorta [**(B,D)**, seven experiments in total] smooth muscle cells isolated from Wistar rats (*n* = 5), treated with increasing concentrations of recombinant mouse chemerin (tested from 5.10^–9^ to 10^–7^ mol/L). Results were expressed as mean ± SEM. *0.01 < *p* < 0.05 and **0.001 < *p* < 0.01 compared to vehicle [0% fetal calf serum (FCS)]-treated smooth muscle cells in the same type of vessels. Representative FACS dot plots and quantification of annexin V/propidium iodide dual labeling in primary cultured pulmonary artery [**(E)**, 14 experiments in total] and thoracic aorta [**(F)**, 13 experiments in total] smooth muscle cells isolated from Wistar rats (*n* = 8). After 24-h serum starvation, smooth muscle cells were treated with staurosporine (10^–6^ mol/L) with or without increasing concentrations of recombinant mouse chemerin (tested from 5.10^–9^ to 10^–7^ mol/L) for 24 h. Percentages of apoptotic cells corresponded to percentages of cells in early and late apoptosis. Results were presented as mean ± SEM. *0.01 < *p* < 0.05, **0.001 < *p* < 0.01 and ***p < 0.001 compared corresponding vehicle (0% FCS)-treated smooth muscle cells; ^###^*p* < 0.001 compared to corresponding staurosporine (10^–6^ mol/L)-treated smooth muscle cells; ^$^*p* < 0.05 and ^$$^0.001 < *p* < 0.01 compared to corresponding chemerin (5.10^–9^ mol/L) and staurosporine (10^–6^ mol/L)-treated smooth muscle cells.

Subsequent experiments were performed to investigate whether the expression of genes encoding for pro- and anti-apoptotic factors were influenced by chemerin in SMCs. We measured mRNA levels of pro-apoptotic Bax and anti-apoptotic Bcl2. No significant differences were found in mRNA expressions of Bax (Figures 4A,B). In contrast, chemerin treatment concentration-dependently increased mRNA Bcl2 levels in both pulmonary artery and thoracic aorta SMCs (Figures 4A,B). Consistently, chemerin (tested from 5.10^–9^ to 10^–7^ mol/L) concentration-dependently decreased the pro-apoptotic Bax-to-Bcl2 ratio in rat pulmonary artery (Figure 4C) and thoracic aorta (Figure 4D) SMCs, suggesting increased resistance to activate apoptotic processes in chemerin-treated SMCs.

We next investigated whether chemerin influenced staurosporine (10^–6^ mol/L)-induced activation of apoptotic processes in pulmonary artery and thoracic aorta SMCs. Chemerin alone (tested at 10^–7^ mol/L) did not alter apoptotic rate in pulmonary artery (Figure 4E) and thoracic aorta (Figure 4F) SMCs. In staurosporine-treated SMCs, chemerin (tested from 5.10^–9^ to 10^–7^ mol/L) decreased apoptotic rate in a concentration-dependent manner in both pulmonary artery (Figure 4E) and thoracic aorta (Figure 4F) SMCs. Staurosporine-induced apoptosis was strongly reduced by 10^–7^ mol/L chemerin treatment. Pulmonary artery and thoracic aorta SMCs responded similarly (Figures 4C,D).

### Chemerin Alone or Associated With Endothelin-1 Induced SMC Migration

**FIGURE 5:**
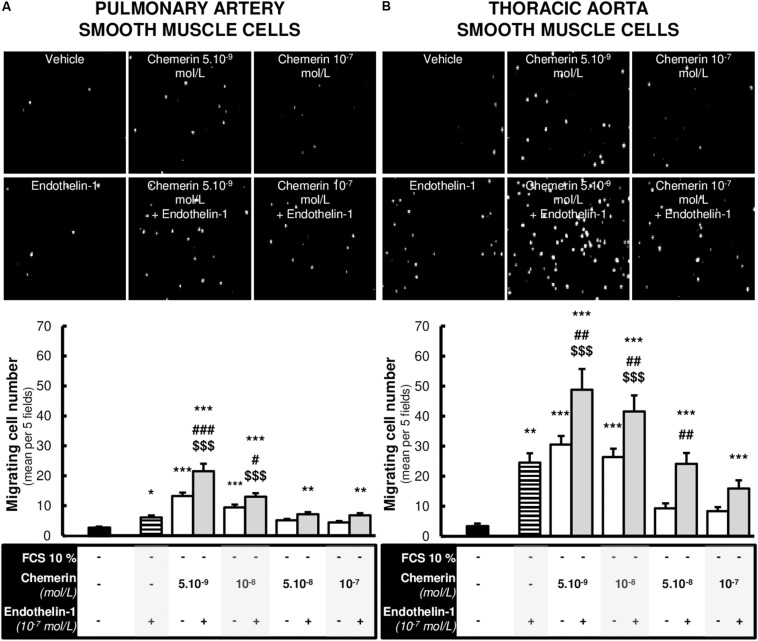
Chemerin-induced migration of pulmonary artery and thoracic aorta smooth muscle cells. Representative contrasted images and quantification of *in vitro* migration of primary cultured pulmonary artery [**(A)**, 12 experiments in total] and thoracic aorta [**(B)**, 9 experiments in total] smooth muscle cells isolated from Wistar rats (*n* = 4), in the modified Boyden exposed or not to recombinant rat endothelin-1 (10^–7^ mol/L) with or without increasing concentrations of recombinant mouse chemerin (tested from 5.10^–9^ to 10^–7^ mol/L) for 24 h. Migration responses were expressed as mean number of migrating cells evaluated in five different fields randomly chosen per insert (at 10× magnitude) and presented as mean ± SEM. *0.01 < *p* < 0.05, **0.001 < *p* < 0.01, ***p < 0.001 compared to basal condition [corresponding to 0% fetal calf serum (FCS)-treated] in the same type of smooth muscle cells; ^#^0.01 < *p* < 0.05, ^##^0.001 < *p* < 0.01 and ^###^*p* < 0.001 compared to corresponding chemerin-treated cells; ^$$$^*p* < 0.001 compared to corresponding endothelin-1-treated cells.

We evaluated the effects of chemerin on SMC migration, a phenomenon that plays a key role in arterial remodeling, such as those encountered in pulmonary arteries in pulmonary hypertension. We therefore studied the effects of increasing concentrations of chemerin (tested from 5.10^–9^ to 10^–7^ mol/L) in the presence or absence of endothelin-1 (10^–7^ mol/L) on *in vitro* migration of rat pulmonary artery and thoracic aorta SMCs. As illustrated in Figures 5A,B, endothelin-1 and lower concentrations of chemerin alone (5.10^–9^ and 10^–9^ mol/L) induced migration of pulmonary artery and thoracic aorta SMCs, while this effect was lost with higher concentrations of chemerin (5.10^–8^ and 10^–8^ mol/L) in both cell types. Combined treatment of chemerin and endothelin-1 increased migration of pulmonary artery (Figure 5A) and thoracic aorta (Figure 5B) SMCs compared with migration observed with any single treatment. This effect was more pronounced in thoracic aorta compared with pulmonary artery SMCs.

### Endothelin-1 Increased the Expression of Chemerin Receptors in Pulmonary Artery SMCs

**FIGURE 6:**
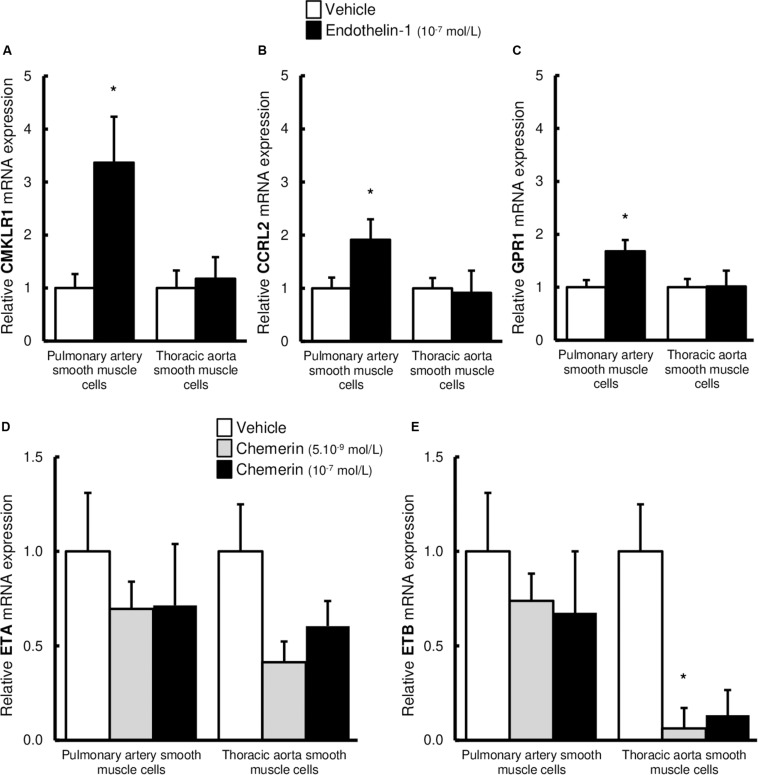
Altered expression of chemerin receptors induced by endothelin-1 in pulmonary artery and thoracic aorta smooth muscle cells. Relative gene expression of chemerin receptors, the chemokine-like receptor 1 [**(A)**, CMKLR1 also called ChemR23], the C-C chemokine receptor-like 2 [**(B)**, CCRL2], and the G protein-coupled receptor 1 [**(C)**, GPR1] in primary cultured pulmonary artery (five experiments in total) and thoracic aorta (seven experiments in total) smooth muscle cells isolated from Wistar rats (*n* = 5). After 24-h serum starvation, smooth muscle cells were treated or not with recombinant rat endothelin-1 (10^–7^ mol/L) for 5 h. Quantification of relative gene expression was achieved by real-time quantitative polymerase chain reaction (RTq-PCR) using the Pfaffl method with normalization with the housekeeping genes, GAPDH and HPRT1. Results were expressed as relative fold increase over the mean value of relative mRNA expression of the vehicle (0% FCS)-treated group arbitrarily fixed to 1 and presented as mean ± SEM. *0.01 < *p* < 0.05 compared to corresponding vehicle-treated condition. Relative gene expression of endothelin receptors type A [**(D)**, ETA] and B [**(E)**, ETB] in primary cultured pulmonary artery (six experiments in total) and thoracic aorta (six experiments in total) smooth muscle cells isolated from Wistar rats (*n* = 5). After 24-h serum starvation, smooth muscle cells were treated with recombinant mouse chemerin (5.10^–9^ and 10^–7^ mol/L) for 5 h. Quantification of relative gene expression was achieved by real-time quantitative polymerase chain reaction (RTq-PCR) using the Pfaffl method with normalization with the housekeeping genes, GAPDH and HPRT1. Results were expressed as relative fold increase over the mean value of relative mRNA expression of the vehicle (0% FCS)-treated group arbitrarily fixed to 1 and presented as mean ± SEM. *p < 0.05 compared to corresponding vehicle-treated condition.

Since we observed that combined treatment with endothelin-1 and chemerin was necessary to promote proliferation in SMCs, we investigated if endothelin-1 (10^–7^ mol/L) could affect expression of chemerin receptors in pulmonary artery and thoracic aorta SMCs. As illustrated in Figures 6A–C, endothelin-1 increased mRNA expression of CMKLR1, CCRL2, and GPR1 in pulmonary artery SMCs, while it did not in thoracic aorta SMCs. In pulmonary artery SMCs, endothelin-1-induced increase in CMKLR1 expression was higher than those observed for CCRL2 and GPR1. After, we also evaluated if chemerin affected mRNA expression levels of endothelin receptors type A (ETA) and B (ETB). In pulmonary artery and thoracic aorta SMCs, chemerin (tested at 5.10^–9^ mol/L and 10^–7^ mol/L) did not alter endothelin receptor (ETA and ETB) mRNA expression, except for ETB expression which was significantly reduced in thoracic aorta SMCs after chemerin treatment (at 5.10^–9^ mol/L) (Figures 6D,E). To evaluate potential role of chemerin on vascular inflammatory phenotype, we evaluated the mRNA expression of inflammatory cytokines. In pulmonary artery SMCs, chemerin did not alter gene expression of interleukin (IL)-6, its receptor IL-6R and IL-1β, while tumor necrosis factor (TNF)-α expression increased with 10^–7^ mol/L chemerin (Supplementary Figure S1A). In thoracic aorta SMCs, treatment with chemerin (10^–7^ mol/L) increased gene expression of IL-6 and IL-1β, while IL-6R and TNF-α expression decreased after both 5.10^–9^ and 10^–7^ mol/L chemerin treatment (Supplementary Figure S1B).

## Discussion

The present study shows that chemerin (alone and/or in presence of endothelin-1) increases *in vitro* proliferation, resistance to apoptosis and migration of rat primary cultured pulmonary artery SMCs. These effects (except for the migration) were stronger than those observed in thoracic aorta SMCs. In pulmonary artery SMCs, endothelin-1 upregulates the gene expression of chemerin receptors, CMKLR1, CCRL2, and GPR1, which could at least partly explain sustained proliferative effects observed when chemerin was combined with endothelin-1.

Three receptors called CMKRL1, CCRL2, and GPR1 have been reported to bind chemerin (Samson et al., 1998; Zabel et al., 2008). CMKLR1 has been mainly associated with chemerin-associated proliferative and chemotactic action, as well as signal transduction (Mattern et al., 2014). Here, we showed that CMKLR1 was expressed in both pulmonary artery intimal and medial layers, with a stronger expression in pulmonary artery SMCs. Previous data showed that CMKLR1 was expressed in both vascular endothelium and SMCs (Watts et al., 2013) and that primary SMCs were able to respond to recombinant chemerin with a calcium flux (Ferland et al., 2017). We also showed the expression of two other chemerin receptors CCRL2 and GPR1 in pulmonary artery SMCs. CCRL2, which is a non-signaling receptor for chemerin (Wittamer et al., 2003; Zabel et al., 2008), may serve as a chemerin membrane anchoring protein that contributes to increased local concentration of chemerin and allows ligand presentation to other chemerin receptors (Zabel et al., 2008; Yoshimura and Oppenheim, 2011; Mazzotti et al., 2017). Through GPR1, chemerin-induced calcium mobilization is only 30% of that induced by CMKLR1 (Barnea et al., 2008; Zabel et al., 2008), and the exact biological role of this receptor remains unclear (Bondue et al., 2011).

The adipokine chemerin is mainly produced by adipose tissue and liver but also by many other tissues and cells, including cardiac, vascular, and pulmonary cells (Wittamer et al., 2003; Goralski et al., 2007). Circulating serum levels of chemerin have been reported around 10^–8^ mol/L in healthy lean rats and humans (Stejskal et al., 2008; Stelmanska et al., 2013) and increased in pathological conditions, such as diabetes and obesity (Zabel et al., 2005a; Lin et al., 2019). We recently showed that chemerin and its three receptors were expressed in both endothelial cells and SMCs in rat pulmonary arteries (Hanthazi et al., 2019). Here, we showed that primary cultured SMCs from rat pulmonary artery conserved *in vitro* the expression of chemerin and its three receptors. Given that chemerin is expressed by pulmonary artery SMCs, we can presume that SMC-derived chemerin could act on pulmonary artery SMCs, in an autocrine/paracrine manner (Ferland and Watts, 2015), without entering the circulation. In addition to local chemerin production, the expression of proteolytic enzymes responsible for the local activation of prochemerin should be investigated in the pulmonary vascular wall (Wittamer et al., 2004; Bondue et al., 2011). Indeed, they would contribute to the production of a chemerin isoform potentially directly active (Zabel et al., 2005b) within the pulmonary vascular wall.

In the present study, we showed for the first time that chemerin, in presence of endothelin-1 increased *in vitro* the proliferation in pulmonary artery SMCs, while chemerin or endothelin-1 alone did not. We choose to test the effects of chemerin in the presence of endothelin-1 which has been largely incriminated in the pathogenesis of pulmonary hypertension and is currently therapeutically targeted to treat the disease (Galie et al., 2004, 2019). Previous work has already presented endothelin-1, as a co-mitogenic factor potentiating growth-promoting effects of other factors (Panettieri et al., 1996) and contributing to pulmonary artery remodeling in pulmonary arterial hypertension (Yang et al., 1999; Maruyama et al., 2015). Here, we failed to find increased proliferation induced by chemerin in thoracic aorta SMCs. This is indeed divergent from previous studies showing that chemerin stimulated rat arterial SMC proliferation and migration (via Akt and ERK activation), as well as carotid intimal hyperplasia and remodeling (Kunimoto et al., 2015; Xiong et al., 2016). Chemerin has also been shown to increase mouse myoblast proliferation and to suppress differentiation acting on ERK signaling (Yang et al., 2012). Neves et al. (2015) showed that chemerin, through redox-sensitive processes, induced proliferation and apoptosis in vascular SMCs. Downregulation of chemerin inhibited vascular SMC proliferation *in vitro*, contributing therefore *in vivo* to reduced neointimal hyperplasia (Xiong et al., 2016). Further investigation revealed that knockdown of chemerin receptor, CMKRL1, inhibited proliferation and migration of vascular SMCs (Liu et al., 2016), suggesting main chemerin-induced activity through CMKLR1. These apparent discrepant results may result from variable cell proliferation protocol and SMC origin, but also to lower concentrations of chemerin compared to those used in previously cited studies. Chemerin concentration used in the present study is within the pathophysiologically proper range (Zabel et al., 2006; Stelmanska et al., 2013; Lin et al., 2019).

Because an imbalance between proliferation and apoptosis is an important feature of pulmonary artery remodeling, we evaluated chemerin regulation of both biological cellular responses. We showed for the first time that chemerin protected against staurosporine-induced apoptosis and decreased pro-apoptotic Bax-to-Bcl2 ratio in pulmonary artery SMCs. Similar results were obtained in thoracic aorta SMCs, which is discrepant compared to previous published data. Indeed, chemerin has been shown to induce apoptosis in different cell types, including cardiomyocytes (Rodriguez-Penas et al., 2015) and vascular SMCs (Neves et al., 2015). We do not have evident explanations for this variation.

Evidence shows that migration of SMCs to the intima and their proliferative capacity are crucial steps in vascular remodeling. In this context, chemerin was also shown to induce migration of pulmonary artery and thoracic aorta SMCs, effect which was increased in presence of endothelin-1. Chemerin has been shown to stimulate chemotaxis in several cell types, including inflammatory cells (Gonzalvo-Feo et al., 2014), as well as cancer (Kumar et al., 2019) and vascular cells (Kunimoto et al., 2015). Chemerin was also able to induce migration of human natural killer cells via activation of ERK (Carlino et al., 2012). Chemerin-induced migration/invasion of bone marrow-derived dendritic cells and macrophages was significantly inhibited in CMKLR1 knockout mice (Luangsay et al., 2009). Furthermore, chemerin enhanced *in vitro* migration, invasion, and proliferation of rat mesenteric artery and human aortic SMCs in a concentration-dependent manner, and the effect of chemerin on SMC migration was largely blocked by CMKRL1 silencing (Kunimoto et al., 2015). Altogether, these data suggest that the effects of chemerin on migration of different cells such as immune and SMCs are mainly dependent on CMKLR1.

We previously showed that chemerin potentiated endothelin-mediated vasoconstriction in pulmonary arteries isolated *in toto* from healthy adult rats (Hanthazi et al., 2019). These data were recently confirmed by Omori et al. showing increased chemerin-9-induced pulmonary artery contraction in experimental pulmonary hypertension (Omori et al., 2020). Similarly, we found that chemerin was a co-mitogenic factor for endothelin-1, necessary to allow proliferation in pulmonary artery SMCs. Our results showed that endothelin-1 increased mRNA expression of CMKLR1, CCRL2, and GPR1 in pulmonary artery SMCs, while chemerin did not alter endothelin receptor levels, suggesting a role played by endothelin-1 as a positive regulator of chemerin/CMKLR1 signaling. Alteration in chemerin signaling has already been linked to other pro-inflammatory conditions or cytokines (including TNF-α, IL-1β, and IL-6) upregulating CMKLR1 (Kaur et al., 2010; Bondue et al., 2011) and CCRL2 (Yoshimura and Oppenheim, 2011; Monnier et al., 2012) which are also known to play crucial roles in pulmonary artery remodeling in pulmonary hypertension. Moreover, this effect was more pronounced for CMKLR1, which is currently known as the main chemerin activity-linked receptor (Omori et al., 2020). Indeed, we know that CCRL2 does not induce the activation of intracellular signaling pathways but regulates chemerin bioavailability (Zabel et al., 2008; Monnier et al., 2012). Endothelin-induced increase in CCRL2 expression could therefore contribute to the accumulation and presentation of chemerin to CMKLR1 in pulmonary artery SMCs. In the present study, expressions of IL-6 and IL-1β were not altered by chemerin in pulmonary artery smooth muscle cells. However, chemerin induced expression of TNF-α, which has been shown to contribute to pulmonary artery SMC remodeling in pulmonary arterial hypertension (Hurst et al., 2017). One limitation of the present study remains, however, that we did not identify precise mechanisms incriminated in these synergic effects, as well as downstream signaling pathways. This should be investigated in future studies, including the precise implication of CMKLR1 and the potential role played by pro-inflammatory cytokines, such as TNF-α. Taken together, our data strongly suggest a role played by endothelin-1 as a positive regulator of chemerin/CMKLR1 signaling.

The present study shows that the adipokine chemerin alone or in association with endothelin-1, induces *in vitro* proliferation, apoptosis resistance, and migration in primary cultured rat pulmonary artery SMCs. Further research is, however, required to better understand the potential role of chemerin and its receptors in the pathophysiologic processes of pulmonary artery remodeling observed in pulmonary hypertension.

## Data Availability Statement

The raw data supporting the conclusions of this article will be made available by the authors, without undue reservation.

## Ethics Statement

The animal study was reviewed and approved by the Institutional Animal Care and Use Committee of the Faculty of Medicine at the Université libre de Bruxelles (Brussels, Belgium; protocol acceptation number: 561N). Applicable guidelines were followed in accordance with the “Guide for the Care and Use of Laboratory Animals” published by the US National Institutes of Health (NIH publication no. 85–23, revised 1996).

## Author Contributions

AH, PJ, LD, and KM conceptualized and designed the study. AH, PJ, GV, CD, and GH performed the research. AH, PJ, J-YS, LD, and KM analyzed the data. AH, PJ, GV, LD, and KM contributed new methods or models. AH, LD, and KM wrote the original draft. All authors have read and agreed to the published version of the manuscript.

## Conflict of Interest

The authors declare that the research was conducted in the absence of any commercial or financial relationships that could be construed as a potential conflict of interest.
