# Supplementary material for: Chemerin Added to Endothelin-1 Promotes Rat Pulmonary Artery Smooth Muscle Cell Proliferation and Migration
Source: Front Physiol. 2020 Jul 30;11:926. doi: 10.3389/fphys.2020.00926 (PMC7406802; doi:10.3389/fphys.2020.00926)
Supplement: Supplementary file 1 [file Table_1.DOCX]

Supplementary Material

**Supplementary Table 1.**

| Genes |  | Sequences | |  |
| --- | --- | --- | --- | --- |
| Interleukin-6 (**IL-6**) | Sense  Antisense | | 5' - CCACCAGGAACGAAAGTCA - 3'  5' - TCAGTCCCAAGAAGGCAACT - 3' | |
| IL6 receptor (**IL-6R**) | Sense  Antisense | | 5' - GGCCACTGTTACCCTGATCT - 3'  5' - TCCCGTGGTAGTCCATTCTC - 3' | |
| Interleukin-1beta (**IL-1β**) | Sense  Antisense | | 5' - AAAAATGCCTCGTGCTGTCT - 3'  5' - TCGTTGCTTGTCTCTCCTTG - 3' | |
| Tumor necrosis factor-alpha (**TNF-α**) | Sense  Antisense | | 5' - ATGGGCTCCCTCTCATCAGT - 3'  5' - GCTTGGTGGTTTGCTACGAC - 3' | |

**
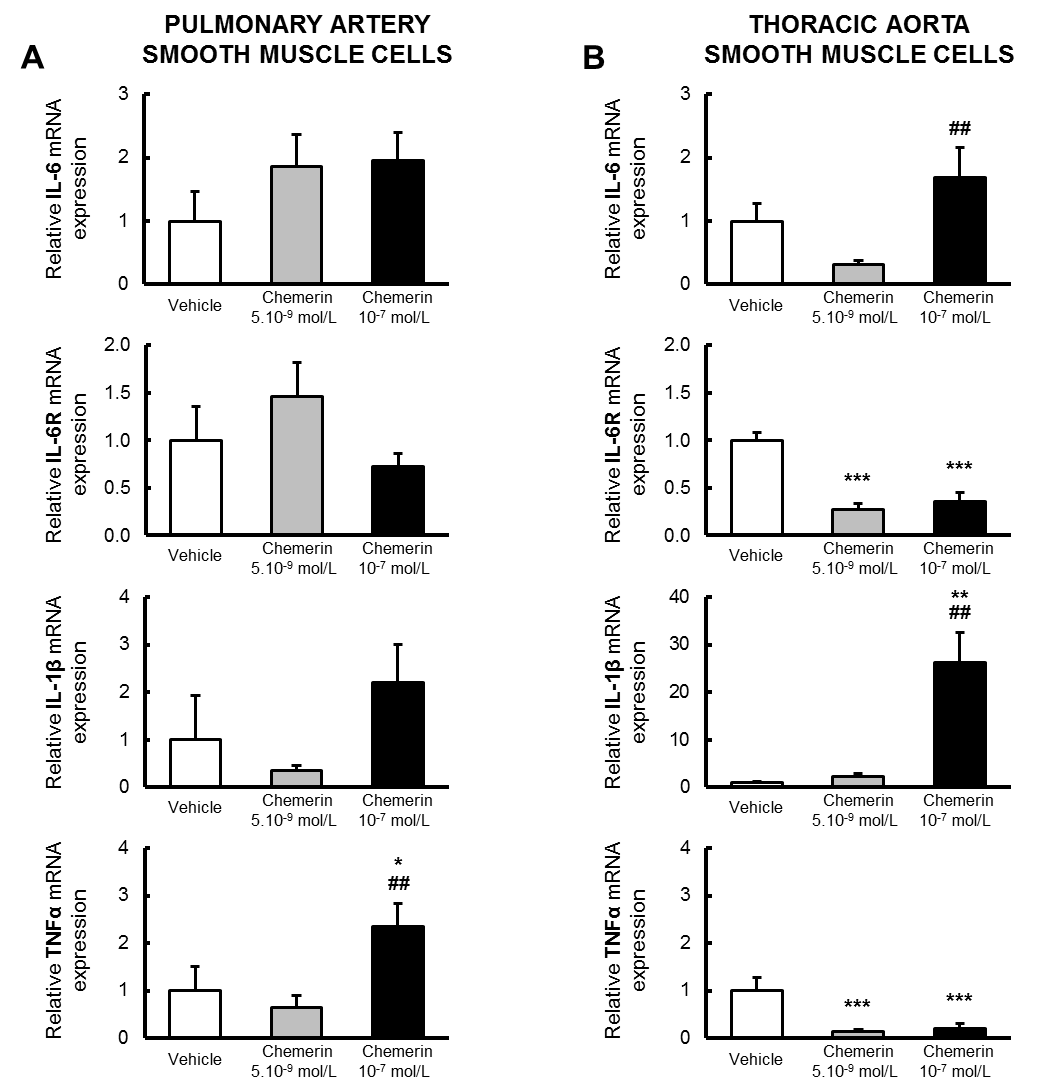
**

**Supplementary Figure 1.** Altered expression of inflammatory markers induced by chemerin in pulmonary artery and thoracic aorta smooth muscle cells. Relative gene expression of interleukin (IL)-6 and its receptor (IL-6R), IL-1β and tumor necrosis factor (TNF)-α in primary cultured vascular smooth muscle cells from Wistar rats (n= 5) in pulmonary artery (**A**; 6 experiments in total) and thoracic aorta (**B**; 6 experiments in total) smooth muscle cells respectively. After 24-hour serum starvation, smooth muscle cells were treated with recombinant mouse chemerin (5.10^-9^ and 10^-7^ mol/L) for 5 hours. Quantification of relative gene expression was achieved by real time quantitative polymerase chain reaction (RTq-PCR) using the Pfaffl method with normalization with the housekeeping genes, glyceraldehyde-3-phosphate deshydrogenase (GAPDH) and hypoxanthine phosphoribosyltransferase (HPRT)1. Results were expressed as relative fold increase over the mean value of relative mRNA expression of the vehicle (0% FCS)-treated group arbitrarily fixed to 1 and presented as mean ± SEM. * 0.01<p< 0.05, ** 0.001<p< 0.01, *** p< 0.001 compared to corresponding vehicle-treated condition; # 0.01<p< 0.05 and ## 0.001<p<0.01 compared to the lower dose of chemerin-treated cells.
